# Sociodemographic factors, anxiety and attitudes toward generative artificial intelligence among nurses

**DOI:** 10.3389/fpsyt.2026.1779382

**Published:** 2026-04-27

**Authors:** Eman Alnazly, Nadine Absy, Nader Absy

**Affiliations:** 1 Faculty of Nursing, Al-Ahliyya Amman University, Amman, Jordan; 2 Medical Education Department, Clinical Teaching Fellow South Tyneside Sunderland National Health Service (NHS) Foundation Trust, Sunderland, United Kingdom; 3 Student at Faculty of Information Technology, Al-Ahliyya Amman University, Amman, Jordan

**Keywords:** Artificial intelligence anxiety, attitudes toward technology, digital health, generative artificial intelligence, healthcare innovation, nursing, sociodemographic factors, technology acceptance

## Abstract

**Background:**

Although generative artificial intelligence offers substantial potential benefits in healthcare, negative attitudes and elevated anxiety among nurses may hinder its effective integration into clinical practice. Evidence regarding the psychological impact of generative artificial intelligence on nurses remains limited.

**Objective:**

This study examined the relationships among sociodemographic characteristics, anxiety, and attitudes toward generative artificial intelligence among nurses.

**Methods:**

A cross-sectional correlational design was employed. Data were collected from 312 hospital nurses using online questionnaires assessing sociodemographic characteristics, attitudes toward artificial intelligence, and artificial intelligence-related anxiety. Data were analyzed using IBM Statistical Package for the Social Sciences (SPSS) Statistics software version 28.

**Results:**

Higher levels of artificial intelligence-related anxiety were associated with less favorable attitudes toward artificial intelligence. Sociodemographic characteristics and anxiety scores collectively explained 49.4% of the total variance in attitudes toward artificial intelligence. Gender, experience with artificial intelligence, use of artificial intelligence in nursing care, awareness of artificial intelligence applications in healthcare, hours spent on the internet, age, and professional experience accounted for 24.7% of the variance in negative attitudes toward generative artificial intelligence.

**Conclusion:**

Anxiety and experiential factors play a central role in shaping nurses’ attitudes toward generative artificial intelligence. Increasing nurses’ exposure to and awareness of artificial intelligence in nursing practice may reduce anxiety and support its acceptance and appropriate use.

## 1 Introduction

Generative artificial intelligence (GenAI) encompasses a broad range of artificial intelligence (AI) systems capable of producing novel content, including text, music, and images. Unlike traditional AI systems, which rely on predefined rules, GenAI generates original outputs by learning from existing data and identifying complex patterns. Its applications span multiple modalities, including text, images, audio, video, three-dimensional (3D) models, and synthetic data (1, 2). In healthcare, GenAI models are increasingly used to provide medical information, support patient consultations, and assist with clinical decision-making (1). The integration of GenAI into healthcare improves diagnostic efficiency and patient outcomes (3) by enabling the analysis of large datasets, including medical images with abnormalities that may be overlooked by humans (4), identifying disease patterns (3), and supporting treatment prediction (4).

AI broadly refers to computer systems designed to perform tasks that typically require human intelligence, such as learning, reasoning, pattern recognition, and the interpretation of complex information (5). While conventional AI systems operate primarily within rule-based frameworks, GenAI can generate new information beyond predictable patterns by leveraging learned representations from large datasets (6). Moreover, unlike traditional AI tools that often require technical expertise, GenAI applications are increasingly accessible and user-friendly, enabling direct use by end users, including healthcare professionals (7).

According to Bajwa et al. (8), AI can enhance patient flow management by efficiently performing administrative tasks, thereby reducing workload intensity for healthcare providers (8). Patients increasingly seek health-related information online, and AI-powered recommendation systems can provide personalized health guidance, support early diagnosis, and facilitate timely access to appropriate healthcare providers (9). Cybersecurity represents another critical application of AI in healthcare, as advanced AI systems can strengthen spyware detection, safeguard sensitive data, and prevents privacy breaches and operational disruptions (10).

The adoption of new technologies can significantly influence nurses’ attitudes, either positively or negatively, depending on their experiences, knowledge, and perceived usefulness (11). Existing literature suggests that healthcare professionals’ attitudes toward AI, including GenAI, generally fall into three categories: high awareness coupled with low usage, mixed positive and negative attitudes, and recognition that targeted interventions can enhance trust in AI systems (1).

Healthcare professionals working with emerging technologies may also experience anxiety, particularly when required to adapt to unfamiliar digital tools. The Technology Acceptance Model (TAM) proposes that people with high anxiety may view AI as less helpful or more challenging to use, which may be related to their views (12, 13). The core constructs of the model include Perceived Usefulness (PU), Perceived Ease of Use (PEOU), Attitude Toward Use (ATT), and Behavioral Intention to Use (BIU) (13). The TAM model has many advantages, but it does not take into consideration previous experience, age, gender, and other personal factors that could affect attitudes towards technology. These personal factors have led to the development of models like the Unified Theory of Acceptance and Use of Technology (UTAUT) (14). The four main components of UTAUT—performance expectancy, effort expectancy, social influence, and facilitating conditions—have a direct impact on the anticipated likelihood of adopting new technology. Gender, age, experience, and voluntariness of use all moderate the important predictors (14).

Knowledge plays a crucial role in healthcare professionals’ attitudes toward AI. Although many nurses reported limited knowledge of AI and its applications, several studies have found that positive attitudes toward AI persist despite this knowledge gap. Nurses’ openness to integrating AI into healthcare practice reflects a willingness to engage with technological innovation, although ethical concerns related to accountability, patient safety, and data privacy remain prominent (15). Similarly, variations in nurses’ levels of AI awareness have been reported, ranging from basic understanding to moderate familiarity; nevertheless, a general willingness to apply AI in practice has been consistently observed (16). Nurses who are active on social media and have experience with AI technology, such as ChatGPT, demonstrated positive attitudes toward the application of AI in healthcare and advocated educating nurses about these technologies. AI has also been identified as a potential facilitator of ongoing professional growth in nursing (17).

In Jordan, the integration of GenAI into the healthcare system remains at an early stage. In contrast to high-income countries, where AI applications are increasingly embedded in healthcare delivery, Jordan faces challenges related to limited digital infrastructure and inconsistent access to advanced technologies (18). These constraints raise concerns regarding the preparedness of Jordanian nurses to engage effectively with AI-driven healthcare systems. Studies indicate that nurses in Jordan encounter substantial challenges, including limited awareness of AI resources, insufficient training opportunities, and a lack of institutional support for digital transformation (18).

### 1.1 Benefits and applications of AI in nursing practice

Current evidence indicates that AI is increasingly being adopted in nursing and is associated with improved efficiency, quality of care, and patient outcomes (18, 19). AI enhances nursing care by reducing diagnostic errors, improving emergency response times, thereby improving overall quality of care, and facilitating remote care, particularly among older adult patients (20).

Implementing comprehensive training programs is essential to equip nurses with the skills necessary for successful AI integration (21, 22) Continuous training and upskilling are essential for the nursing workforce to effectively utilize and interpret AI tools (23), while leaders’ readiness for AI integration and development of policies regarding AI integration in nursing practice Kotp et al. (24) is essential. Another issue that can improve the integration of AI into healthcare is a nursing curriculum reform to prepare nurses for the safe and effective utilization of AI, including education regarding its application and evaluation (25).

### 1.2 Barriers to AI integration in nursing practice

Although the use of AI is associated with several positive nursing outcomes, including improvements in response times and quality of care, some studies have indicated barriers to its integration and risks such as AI anxiety and negative attitudes (26, 27). Attitudes toward AI were strongly and positively correlated with the intent to stay (Oweidat et al. (28). GenAI anxiety refers to the fear individuals exhibit regarding the rapid development and widespread application of GenAI anxiety, which is often caused by concerns that this technology can be utilized unethically, disrupt jobs, or spread false information (29). Notably, Tarsuslu et al. (26) noted a significant and inverse relationship between AI anxiety and attitude. This implies that nurses’ attitudes toward AI influence their anxiety levels. It was further observed that, although most nurses were aware of the use of AI in healthcare, they exhibited medium to high anxiety levels when examined for AI anxiety.

Similarly, Wang and Wang (30) analyzed the development and validation of an AI anxiety scale for predicting motivational behavior, and found that technology had disruptive effects and could lead to concerns regarding skill enhancement or career changes, resulting in anxiety about AI development. Additionally, they noted that there were no valid and reliable scales for measuring AI anxiety among employees despite the increasing levels of AI-related anxiety. They further asserted that traditional instruments for measuring computer or Internet anxiety are inadequate for measuring AI anxiety. Similarly, Wen et al. (27) noted that increasing the adoption of AI technology resulted in rising AI anxiety levels. These findings exhibit that AI anxiety poses more unpredictable risks to individual’s retention of their occupations than computer anxiety because such technology can function autonomously and independently. Wen et al. (27) implied that AI anxiety primarily results from worry and fear that technology could replace or affect future employability. Collectively, these studies exhibit that nurses’ attitudes toward AI influence their anxiety levels, highlighting the need to integrate valid and reliable measurement scales and psychological interventions to enhance their attitudes toward AI tools and reduce their anxiety levels (27). Anxiety and acceptance served as dual mediators in the association between perception and intention to utilize AI among nursing students. Developing AI as part of nursing education enhances students’ attitudes toward it (31).

### 1.3 GenAI and mental health

Several studies have examined the effects of the GenAI on nurses’ mental health. Sallam et al.’s (32) study exhibited that students with higher general anxiety about AI scored higher on fear, anxiety, and ethical concerns. Prior exposure to and use of generative AI did not significantly influence student perceptions (32).

In a different qualitative study, Bodur et al. (33) observed that most nurses reported their anxiety in relation to data breaches and vulnerability to patient information in AI systems. Nirgiz et al. (34) observed that nurses had moderate levels of anxiety but generally positive attitudes toward AI. Male nurses reported lower levels of anxiety and more positive attitudes than female nurses. Nurses with 0–3 years of work experience exhibited lower anxiety and more favorable perceptions of AI (34).

It is established that generative artificial intelligence (GenAI) has a significant impact on healthcare professionals; however, its application in the workplace has been associated with increased anxiety and negative attitudes. Despite this, there remains a gap in literature, particularly within nursing, regarding the relationship between mental health and the use of GenAI.

This study therefore aimed to examine the relationships between sociodemographic characteristics, anxiety, and attitudes toward GenAI among nurses. Specifically, it explored how nurses’ demographic factors relate to their levels of anxiety and their attitudes toward the use of GenAI in healthcare, as well as which sociodemographic variables may predict these outcomes.

## 2 Methodology

### 2.1 Study design

This study employed a cross-sectional quantitative design to examine the relationship between anxiety and nurses’ attitudes toward GenAI.

### 2.2 Participants

Data were collected from nurses employed in hospitals in Amman, Jordan, in December 2024. Participants were recruited through social media platforms. Eligible participants were nurses aged 21 years and older, employed for at least 20 hours per week, with a minimum of six months of professional nursing experience, and holding a Bachelor of Science in Nursing degree. Nurses holding doctorate degrees in nursing or enrolled in doctoral programs were excluded from the study.

### 2.3 Sample size and recruitment

The required sample size was determined using G* Power analysis software (35). Based on a statistical power of 80%, a significance level of 0.05, an effect size of 0.15, and 13 predictors, the minimum required sample size for multiple linear regression analysis was 144 participants. This sample size was sufficient to detect meaningful associations between nurses’ attitudes toward AI and the predictor variables.

Participants were recruited by contacting professional nursing groups through social media platforms, including Facebook, X, and WhatsApp. Relevant accounts were tagged to maximize visibility. Recruitment posts provided information regarding the study purpose, eligibility criteria, possible risks, participants’ rights, estimated completion time, and the researcher’s contact information.

### 2.4 Data collection

Data were collected using an online, self-administered questionnaire in English, developed using Google Forms. The survey was administered between November 30^th^ and December 30^th^, 2024. The questionnaire link was distributed through email and social media platforms. The first section of the questionnaire collected sociodemographic information, including age, educational level, clinical unit, daily internet use, perceptions of AI, and knowledge of AI applications. Participants’ prior experiences with AI were also assessed (**Table 1**). The survey further included the General Attitudes toward AI Scale (GAAIS) and the Artificial Intelligence Anxiety Scale (AIAS).

**Table 1:** Sociodemographic characteristics of the study sample (N = 312).

Variables	Categories	Frequency	Percentage	Mean (SD)
Gender	Male Female	144 168	46.2 53.8	
Education level	Bachelor’s degree Master’s degree	241 71	77.2 22.8	
Type of unit working	Critical General *Others	160 120 32	51.3 38.5 10.3	
Experience with Artificial Intelligence	Yes No	177 135	56.7 43.3	
Used Artificial Intelligence in nursing care such as patient education	Yes No	141 171	45.2 54.8	
Awareness of Artificial Intelligence application in health care	Yes No	203 109	65.1 34.9	
Hours spent on the internet				4.23 (2.09)
Years of experience in nursing				10.60 (6.54)
Age or years				35.37 (7.19)

*Others: Nurses working in radiography, MRI, and non-invasive cardiovascular diseases.

### 2.5 Instruments

#### 2.5.1 The general attitudes toward artificial intelligence scale

The GAAIS was used to assess nurses’ attitudes toward AI. The scale comprises 20 items measuring both positive and negative attitudes. Responses were recorded on a five-point Likert scale ranging from “strongly disagree” to “strongly agree.” Higher scores indicate more positive attitudes toward the application of AI in healthcare. Items assessing negative attitudes were reverse scored. Mean scores were calculated separately for the positive and negative subscales, as the scale developers do not recommend computing an overall score. Higher mean scores on each subscale reflect more positive attitudes. The original scale demonstrated good internal consistency, with Cronbach’s alpha coefficients (α) of 0.88 for the positive subscale, and 0.83 for the negative subscale (36).

#### 2.5.2 The artificial intelligence anxiety scale

The AIAS comprises 21 items designed to assess anxiety related to AI (30). Items are rated on a seven-point Likert-type scale ranging from 1 (never) to 7 (completely). The scale comprises four subscales: learning anxiety, job replacement anxiety, sociotechnical blindness, and AI configuration anxiety. Total scores range from 21 to 147 (37). The scale has demonstrated excellent reliability, with an overall Cronbach’s α of 0.96. Subscale reliability coefficients were α = 0.89 for the Learning Anxiety Subscale (8 items), α = 0.95 for the Job Replacement Anxiety Subscale (6 items), α = 0.89 for the Sociotechnical Blindness Subscale (4 items), and α = 0.95 for the AI Configuration Anxiety Subscale (3 items) (37).

A pilot study was conducted to test the feasibility and practicality of these instruments utilized in this study. The author distributed 26 paper-based questionnaires of the GAAIS and AIAS to nurses working in hospitals who were excluded from the main study. Cronbach’s alpha for the GAAIS was α = 0.81 and α = 0.84 for the AIAS. Test-retest reliability was assessed over a two-week interval with the same participants. Cronbach’s alpha for the GAAIS was α = 0.82 and 0.83 for the AIAS, indicating acceptable reliability.

### 2.6 Data management

Following data collection, responses were exported from Google Forms to Microsoft Excel and subsequently imported into the SPSS version 29 for analysis. There were no missing data because each item on the questionnaire had an asterisk indicating that participants could not submit the form prior to responding to all questions. Subsequently, an analysis was conducted to determine the nurses’ attitudes toward AI. Access to data was restricted to ensure confidentiality. All identifiers were removed during the export of data from Google Forms.

### 2.7 Ethical considerations and confidentiality measures

This study was approved by the Institutional Review Board of Amman Al-Ahliyya University (#MM: 3/01-2025). The participants were informed of the purpose of the study, and their participation was voluntary. Participants were advised that submission of the completed questionnaire implied informed consent. Participant anonymity and confidentiality were ensured. No incentives or rewards were provided, and participants were informed of potential inconveniences associated with participation.

### 2.8 Data analysis

Data analysis included both descriptive and inferential statistics. Descriptive statistics were used to summarize the sociodemographic data, attitudes, and anxiety of the participants toward the AI-integrated tool. For the descriptive analysis, graphs and descriptive tables with means, standard deviations, frequencies, and percentages were generated. Inferential statistics were analyzed using the independent sample *t*-test and one-way ANOVA. These tests were used to investigate the mean differences in nurses’ attitudes toward AI. Pearson’s correlation coefficient was used to explore the relationship between nurses’ attitudes and anxiety. Variables significantly associated with nurses’ attitudes were entered into a multiple linear regression analysis to elucidate their predictions. SPSS IBM software version 28 was used for data analysis. The alpha level was set at 0.05, and p-value less than alpha were considered statistically significant.

## 3 Results

**Table 1** summarizes the sociodemographic characteristics of the participants. The sample comprised 312 nurses, including168 males (46.2%) and 144 females (53.8). Most participants held a bachelor’s degree in nursing (n = 241, 77.2%), while 71 (22.8%) held a master’s degree. A total of 160 nurses (51.3%) worked in critical care units, 120 (38.5%) in general units, and 32 (10.3%) in other departments (e.g., radiology [X-ray], magnetic resonance imaging [MRI], and noninvasive cardiovascular units). More than half of the participants (n = 177, 56.7%) reported prior experience with AI; however, only 141 (45.2%) had used AI in nursing care, such as for patient education. Awareness of AI applications in healthcare was relatively high, with 203 participants (65.1%) reporting familiarity. On average, nurses spent 4.23 hours per day on the internet (*SD* = 2.09). The mean age of participants was 35.37 years (SD = 7.19 years), and the mean duration of nursing experience was 10.60 years (SD = 6.54) (**Table 1**).

### 3.1 Relationship between nurses’ demographics, AI anxiety, and attitudes of nurses toward GenAI

The correlations between nurses’ demographic variables, AI anxiety, and positive and negative attitudes toward GenAI are presented in **Table 2**. The analysis exhibited that male nurses (M = 3.53, SD = 0.66) reported significantly higher positive attitude scores toward AI than female nurses (M = 3.05, SD = 0.62, *P* < 0.001). Moreover, those working in general units (M = 3.39, SD = 0.72) obtained significantly higher attitude scores than those working in other units (M = 3.04, SD = 0.60, LSD p =0.018).

**Table 2:** Relationships between nurses’ demographics characteristics, AI anxiety, and attitudes toward generative AI (GenAI).

Variables	Categories	# Positive GAAIS		*Negative GAAIS	
		Mean	SD	Mean	SD
Gender	Male Female	3.53 3.05	0.66 0.62	2.95 2.46	0.75 0.65
	Test value (*P-value*)	6.617 ***(<0.001***) *^t^*		6.182 (<0.001) *^t^*	
Type of unit working	Critical General Others	3.23 3.39 3.04	0.56 0.72 0.60	2.70 2.64 2.86	0.64 0.73 0.85
	Test value (*P-value*)	4.574 (***0.011***) *^F^*		1.309 (*0.271*) *^F^*	
Education level	Bachelor’s degree Master’s degree	3.31 3.17	0.66 0.57	2.71 2.63	0.71 0.64
	Test value (*P-value*)	1.613 (*0.108*) *^t^*		0.816 (*0.415*) *^t^*	
Do you have experience with Artificial Intelligence	Yes No	3.40 3.10	0.64 0.63	3.01 2.22	0.72 0.66
	Test value (*P-value*)	4.130 ***(<0.001***)*^t^*		9.951 ***(<0.001****) ^t^*	
Have you used Artificial Intelligence in nursing care such as patient education	Yes No	3.45 3.12	0.68 0.60	2.81 2.59	0.73 0.67
	Test value (*P-value*)	4.551 ***(<0.001***)*^t^*		2.771 (***0.005***)*^t^*	
Are you aware about Artificial Intelligence application in health care	Yes No	3.33 3.16	0.66 0.60	2.76 2.57	0.73 0.63
	Test value (*P-value*)	2.237 (***0.025***) *^t^*		2.296 (***0.022***) *^t^*	
Hours spent on the internet	Test value (*P-value*)	0.225 *(****<0.001***) ^r^		0.163 *(****0.004***) ^r^	
Years of experience in nursing	Test value (*P-value*)	-0.123 (***0.030***) ^r^		-0.126 (***0.028***) ^r^	
Age or years	Test value (*P-value*)	-0.121 (***0.032***) ^r^		-0.115 (***0.030***) ^r^	
Artificial intelligence anxiety					
AI learning anxiety	Test value (*P-value*)	-0.163 (***0.004)*** ^r^		-0.490 (<0.001) ^r^	
Job replacement anxiety	Test value (*P-value*)	-0.249 (<0.001) ^r^		-0.477 (<0.001) ^r^	
Sociotechnical blindness	Test value (*P-value*)	-0.264 (<0.001) ^r^		-0.388 (<0.001) ^r^	
AI configuration anxiety	Test value (*P-value*)	-0.267 (<0.001) ^r^		-0.399 (<0.001) ^r^	

^t^independent t-test, ^F^one-way ANOVA, ^r^Pearson correlation.

* Higher score on negative GAAIS domain indicates to have more forgiving about the negative attitudes due to reversing score.

^#^ Higher score on positive GAAIS domain indicates to have higher positive attitude score.

The bold indicated P value <0.05, means statistically significant.

Nurses with experience in AI (M = 3.40, SD = 0.64), those who had used AI in nursing care (M = 3.45, SD = 0.68), and those with awareness regarding AI application in healthcare (M = 3.33, SD = 0.66) reported significantly higher attitudes toward GenAI compared to those without experience (M = 3.10, SD = 0.63), those who had never used it in nursing care (M = 3.12, SD = 0.60), and those lacking awareness about its application (M = 3.16, SD = 0.60). All mean differences were statistically significant (*P* < 0.05).

The number of hours spent on the internet was positively correlated with the positive attitude domain toward GenAI (*r* = 0.225, *P* < 0.001). Nurses’ age (*r* = 0.121), experience (*r* = 0.123), AI learning anxiety (*r* = 0.163), job replacement anxiety (*r* = 0.249), sociotechnical blindness (*r* = 0.264), and AI configuration anxiety (*r* = 0.267) were negatively correlated with the positive attitude domain toward the GAAIS, all of which were significant (*P* < 0.05).

In the negative domain of the GAAIS, the scores are reversed; therefore, higher scores indicate greater leniency or forgiveness regarding negative attitudes. Male nurses (M = 2.95, SD = 0.75) reported significantly higher lenience scores in this domain than female nurses (M = 2.46, SD = 0.65*, P<*0.001). Additionally, nurses with experience in AI (M = 3.01, SD = 0.72), those who had used AI in nursing care (M = 2.81, SD = 0.73), and those with awareness of AI applications in healthcare (M = 2.76, SD = 0.73) reported significantly more forgiving attitudes toward the negative aspects of GenAI than those without experience (M = 2.22, SD = 0.66), those who had never used it in nursing care (M = 2.59, SD = 0.67), and those lacking awareness of its application (M = 2.57, SD = 0.63). All mean differences were significant (*P* < 0.05).

Greater internet use was positively associated with leniency toward negative attitudes (*r* = 0.163, *P* = 0.004). Conversely, older nurses (*r* = -0.115), longer professional experience (*r* = -0.126), those experiencing high levels of AI learning anxiety (*r* = -0.490), job replacement anxiety (*r* = -0.477), sociotechnical blindness (*r* = -0.388), and AI configuration anxiety (*r* = -0.399) were significantly correlated with less forgiving of negative attitudes toward the GAAIS. All correlation coefficients were significant (*P* < 0.05; **Table 2**).

### 3.2 Predicting positive GAAIS scores using hierarchical multiple linear regression

Hierarchical multiple linear regression analysis was conducted to examine predictors of positive attitudes toward GenAI. In Model 1, nurses’ gender, unit of work, experience with AI, use of AI in nursing care, awareness of AI applications in healthcare, hours spent on the internet, age, and professional experience explained 38.6% of the variance in positive attitudes toward GenAI (*F=22.719, P<0.001*). Being a male (*B* = 0.171, *P* = 0.007), having experience with AI (*B* = 0.286, *P* < 0.001), having used AI in nursing practice (*B* = 0.123, *P* = 0.044), awareness of AI applications in healthcare (*B* = 0.143, *P* = 0.034), and greater internet use (*B* = 0.055, *P* < 0.001) significantly predicted more positive attitudes toward GenAI. Age was a significant inverse predictor (B = 0.017, P = 0.001). Professional experience and unit of work were not significant predictors in this model (**Table 3**).

**Table 3:** Predictors of positive GAAIS scores based on hierarchical multiple linear regression.

Variables	*Model 1*					*Model II*				
	*B*	*SE*	*β*	*t*	*p*	*B*	*SE*	*β*	*t*	*p*
Demographics										
Gender (males)	0.171	0.063	0.134	2.702	***0.007***	0.151	0.058	0.118	2.600	***0.010***
Do you have experience with Artificial Intelligence	0.286	0.064	0.224	4.473	***0.001***	0.278	0.058	0.218	4.762	***0.001***
Have you used Artificial Intelligence in nursing care such as patient education	0.123	0.061	0.096	2.019	***0.044***	0.150	0.056	0.117	2.676	***0.008***
Are you aware about Artificial Intelligence application in health care	0.143	0.067	0.110	2.130	***0.034***	0.142	0.061	0.108	2.318	***0.021***
Hours spent on the internet	0.055	0.015	0.183	3.582	***0.001***	0.051	0.014	0.168	3.590	***0.001***
Age in years	-0.017	0.005	-0.216	-3.239	***0.001***	-0.018	0.005	-0.220	-3.582	***0.001***
Years of experience in nursing	-0.009	0.007	-0.084	-1.202	0.230	-0.005	0.007	-0.046	-0.706	0.481
Critical working unit	0.135	0.098	0.106	1.374	0.170	0.116	0.089	0.091	1.296	0.196
General working unit	0.157	0.101	0.120	1.552	0.122	0.135	0.093	0.103	1.459	0.146
AI anxiety										
AI learning						-0.077	0.022	-0.153	-3.525	***0.001***
Job replacement						-0.058	0.028	-0.125	-2.073	***0.039***
Sociotechnical blindness						-0.064	0.031	-0.138	-2.089	***0.038***
AI configuration						-0.058	0.029	-0.129	-2.009	***0.045***
R^2^	0.404					0.515				
Adjusted R^2^	0.386					0.494				
Adjusted R^2^ change	0.404					0.111				
F for Change in R^2^	22.719^**^					17.090^**^				

The bold indicated P value <0.05, means statistically significant. **p < 0.001.

In Model 2, the inclusion of AI anxiety subscales explained an additional 11.1% of the variance (F change =17.090, *P* < 0.001). AI learning anxiety (*B* = -0.077), job replacement anxiety (*B* = -0.058), sociotechnical blindness (*B* = -0.064) and AI configuration anxiety (*B* = -0.058) were all significant inverse predictors toward GenAI (*P* < 0.05). Collectively, nurses’ demographics characteristics and AIAS subscales explained 49.4% of the total variance in positive attitudes (**Table 3**).

### 3.3 Predicting the negative GAAIS scores using hierarchical multiple linear regression

Hierarchical multiple linear regression was also conducted to examine predictors of negative attitudes toward generative artificial intelligence. In Model 1, nurses’ gender, experience with AI, use of AI in nursing care, awareness of AI application in healthcare, hours spent on the internet, age, and profession experience explained 24.7% (R^2adj^=0.247) of the variance in negative attitudes toward GenAI (*F=14.261, P<0.001*). Being a male nurse (*B* = 0.233, *P* = 0.002), having experience with AI (*B* = 0.703, *P* = 0.001), having used AI in nursing practice (*B* = 0.205, *P* = 0.010), awareness of AI application in healthcare (*B* = 0.179, *P* = 0.037), and greater internet use (*B* = 0.046, *P* = 0.014) were significantly associated with greater leniency or forgiveness of negative attitudes toward GenAI. This indicates that nurses who are familiar with GenAI have increased tolerance or leniency about negative attitudes toward GenAI technology. Age and professional experience were not significant predictors in this model (**Table 4**).

**Table 4:** Predictors of negative GAAIS scores based on hierarchical multiple linear regression.

Variables	*Model 1*					*Model II*				
	*B*	*SE*	*β*	*t*	*p*	*B*	*SE*	*β*	*t*	*p*
Demographics										
Gender (Males)	0.233	0.074	0.164	3.169	***0.002***	0.224	0.063	0.157	3.523	***0.001***
Do you have experience with Artificial Intelligence	0.703	0.083	0.503	8.449	***0.001***	0.521	0.074	0.373	7.025	***0.001***
Have you used Artificial Intelligence in nursing care such as patient education	0.205	0.079	0.146	2.576	***0.010***	0.251	0.069	0.179	3.622	***0.001***
Are you aware about Artificial Intelligence application in health care	0.179	0.086	0.121	2.091	***0.037***	0.145	0.074	0.098	1.971	0.052
Hours spent on the internet	0.046	0.019	0.138	2.482	***0.014***	0.028	0.016	0.085	1.751	0.081
Years of experience in nursing	-0.016	0.023	-0.149	-0.701	0.484	-0.019	0.020	-0.181	-0.984	0.326
Age in years	-0.023	0.020	-0.238	-1.129	0.260	-0.021	0.018	-0.220	-1.210	0.227
AI anxiety										
AI learning						-0.060	0.026	-0.113	-2.322	***0.021***
Job replacement						-0.089	0.032	-0.174	-2.798	***0.005***
Sociotechnical blindness						-0.067	0.033	-0.128	-2.012	***0.045***
AI configuration						-0.091	0.033	-0.174	-2.745	***0.006***
R^2^	0.247					0.454				
Adjusted R^2^	0.230					0.434				
Adjusted R^2^ change	0.247					0.207				
F for Change in R2	14.261^**^					28.363^**^				

The bold indicated P value <0.05, means statistically significant. **p < 0.001.

In Model 2, the inclusion of AI anxiety subscales explained an additional 20.7% (R^2adj^=0.207) of the variance (*F* change =28.363, *P* < 0.001). AI learning anxiety (*B* = -0.060), job replacement anxiety (*B* = -0.089), sociotechnical blindness (*B* = -0.067) and AI configuration anxiety (*B* = -0.091) were significant predictors of negative attitudes toward AI. Higher AI anxiety scores were associated with lower leniency or forgiveness of negative attitudes toward GenAI. All correlation coefficients were significant (*P* < 0.05) and nurses’ demographics as well as AI subscales collectively explained 43.4% (R^2adj^=0.434) of the total variance (**Table 4**).

## 4 Discussion

The findings of this study are largely consistent with prior research. Similar to the findings reported by Alruwaili et al. (15), an inverse relationship was observed between nurses’ age and attitudes toward AI, with younger nurses demonstrating significantly more positive attitudes than older nurses (15). Gender differences were also evident, as female nurses reported significantly more negative attitudes toward AI than male nurses (15). Sumengen et al. (38) found that male nursing students had significantly more positive attitudes toward AI, based on the GAAIS scale, than female students. There is a positive association between AI awareness, AI usage, AI evaluation, and the positive domain of the GAAIS (38). Similar to the findings of this study, Tarsuslu et al. (26) found a significant negative association between AI anxiety and attitude. However, unlike this study, Alruwaili et al. (15) observed that nurses’ years of experience did not influence their attitudes toward AI.

All forms of anxiety (AI learning anxiety, job replacement anxiety, sociotechnical blindness, and AI configuration anxiety) were negatively associated with nurses’ positive attitudes toward AI. According to Uçar, Çapuk, and Yiğit (39), the notion that individuals will lose their jobs because of AI is among the recent concerns regarding AI. There were statistically significant differences in participants’ scores for anxiety toward AI and unemployment anxiety according to gender, trust in technology, and use of AI (39). Additionally, there is a positive association between unemployment and AI anxiety (39). Asio and Suero (40) found a significant relationship between AI anxiety, self-efficacy, and self-competence. Therefore, higher levels of AI anxiety among nurses may discourage their use of AI in nursing practice. Additionally, greater awareness of AI was associated with more positive attitudes toward AI. Özbek et al. (41) found that AI readiness was inversely and significantly associated with AI anxiety among medical students. Collectively, these findings highlight the importance of preparing students to embrace AI in their educational curricula.

The findings of this study indicated that higher levels of AI anxiety among nurses may discourage their use of AI technology in nursing practice. Based on TAM and UTAUT, anxiety has been connected to reduced behavioral control, perceived usefulness, and ease of use, all of which are important variables associated with attitudes and behavioral intentions regarding the use of technology (6, 12, 13). In this study, anxiety related to learning about AI, job displacement anxiety, sociotechnical blindness, and AI configuration anxiety were significantly less forgiving of the negative attitude domain of general attitudes toward AI. When new technology is introduced into healthcare settings, nurses often experience anxiety, which is related to its effectiveness, patient safety, data privacy, and lack of knowledge regarding how to operate the programs (6). Also, nurses who were engaged in social media or online courses have more tolerance or leniency about negative attitudes toward GenAI technology acceptance. This increases confidence and enhances digital literacy, leading to positive attitudes and reduced anxiety. AI technology is perceived as an enhancement tool rather than a work replacement, reflecting the influence of social media on nurses’ acceptance of GenAI and the integration of technology in the delivery of patient care.

The integration of AI into healthcare is associated with various benefits. For instance, AI enables a proactive approach to healthcare through its capabilities in identifying illnesses, customizing care, real-time tracking of patient issues, and anticipating patient needs (42). Ensuring that nurses have positive attitudes and less anxiety toward AI is essential for supporting the adoption and use of AI in nursing practice to optimize its associated benefits.

Tarsuslu et al. (26) found a significant negative association between digital leadership and AI anxiety. Tarsuslu et al. (26) indicated that AI anxiety may be decreased by improving digital leadership. Therefore, the high levels of AI anxiety reported by the participants in this study may be addressed by engaging in appropriate digital leadership practices. Tarsuslu et al. (26) found a significant positive relationship between digital leadership and the attitude toward AI. Nurses’ attitudes toward AI can be improved by adopting appropriate digital leadership practices. The use of the GenAI among hospitals in Jordan differs greatly, which could be a factor in nurses’ anxiety and positive attitudes. Adoption rates and implementation strategies depend on the leadership, knowledge, resources, infrastructure, and focus of each institution.

In this study, nurses’ gender, nursing experience in AI, using AI in nursing care, awareness of AI applications in healthcare, number of hours spent on the Internet, age, and professional experience could only account for 24.7% of the negative attitudes toward GenAI variance, exhibiting greater leniency toward positive attitudes toward GenAI. Higher AI anxiety scores were associated with lower leniency or forgiveness of negative attitudes toward GenAI. As AI technology shapes healthcare, it is important to minimize the gap between hospital nurses’ attitudes and anxiety.

### 4.1 Implications

The findings of this study have several implications for the nursing practice. The findings of the study have exhibited a positive association between nurses attitudes toward AI and their AI experience, awareness, and internet use underscore the importance of increasing nurses’ exposure to more AI in clinical settings. Additionally, age was inversely associated with nurses’ AI attitudes toward AI, highlighting the need for more emphasis on educating older nurses about the importance and use of AI in nursing practice.

The different levels of AI anxiety reported by the nurses in this study may be utilized as motivation to learn about AI and improve their attitudes. It is essential to assess and implement appropriate strategies to decrease nurses’ levels of AI anxiety and encourage their adoption and use to improve the care delivery process as well as patient outcomes.

In this study, nurses who were familiar with AI technology and had more experience using it may have experienced less fear and anxiety toward AI. Anxiety predicts negative attitudes. Participants worked in different hospitals, used different technologies, and were exposure to GenAI, with varying organizational support being provided by the hospitals. Therefore, higher levels of AI anxiety among nurses may discourage their use of AI technology in nursing practice. It is essential to assess and implement appropriate strategies to decrease nurses’ levels of AI anxiety and encourage their adoption and use to improve the care delivery process as well as patient outcomes. Further research is needed to investigate nurses’ emotional relationships with GenAI using longitudinal data and evidence on how to reduce GenAI-related anxiety.

### 4.2 Strength and limitation

This study contributes to the growing body of literature examining the impact of GenAI on healthcare professionals’ mental health. A key strength of this study lies in its comprehensive examination of the relationships among nurses’ demographic characteristics, AI anxiety, and attitudes toward GenAI. The findings provide valuable insights into how sociodemographic factors which were significantly associated with greater leniency or forgiveness about negative attitudes and anxiety collectively influence nurses’ acceptance of AI technologies in healthcare settings.

Nevertheless, several limitations should be acknowledged. The extent of GenAI implementation varied across participating hospitals, influenced by factors such as cybersecurity infrastructure, availability of AI specialists, internet connectivity, and financial resources. Cultural resistance and limited public trust in AI systems may have further complicated adoption (43). The use of convenience sampling may limit the representativeness of the sample or restrict generalizability. Recruitment through social media platforms may have excluded nurses with limited online engagement, and the sample lacked diversity in academic qualifications and representation from rural healthcare settings. Future studies should employ probability-based sampling methods, include a broader range of healthcare professionals, and incorporate diverse geographic and institutional contexts to enhance generalizability. Longitudinal and mixed-methods designs are also recommended to deepen understanding of nurses’ evolving attitudes and emotional responses to GenAI.

### 4.3 Conclusions

This study examined the relationship between anxiety and attitudes toward AI among nurses. The findings indicate that nurses’ experience with AI, awareness of AI applications, and number of hours spent on the internet were positively correlated with positive attitudes toward AI. In contrast, increasing age, professional experience, AI learning anxiety, job replacement anxiety, sociotechnical blindness, and AI configuration anxiety were negatively associated to positive attitudes toward AI. These sociodemographic factors may hinder nurses’ adoption of AI technologies by reinforcing apprehension and limiting confidence in their use. To address these challenges, nursing leaders and healthcare organizations should adopt a proactive role by acknowledging the negative outcomes of deploying GenAI and take further steps to prioritize adopting new technology. Enhancing digital literacy, strengthening technical competencies, and supporting nurses’ emotional preparedness through targeted education and training initiatives may help reduce AI-related anxiety and promote more positive attitudes toward AI in clinical practice.

## Data Availability

The raw data supporting the conclusions of this article will be made available by the authors, without undue reservation.
